# Invasiveness and metastasis of retinoblastoma in an orthotopic zebrafish tumor model

**DOI:** 10.1038/srep10351

**Published:** 2015-07-14

**Authors:** Xiaoyun Chen, Jian Wang, Ziquan Cao, Kayoko Hosaka, Lasse Jensen, Huasheng Yang, Yuping Sun, Rujie Zhuang, Yizhi Liu, Yihai Cao

**Affiliations:** 1 The State Key Laboratory of Ophthalmology, Zhongshan Ophthalmic Center, Sun Yat-Sen University, Guanzhou, 510060, People’s Republic of China; 2 Department of Microbiology, Tumor and Cell Biology, Karolinska Institute, 171 77 Stockholm, Sweden; 3 Department of Oncology, Jinan Central Hospital, Shandong University, NO.105, Jiefang Road, Jinan, Shandong 250013, People’s Republic of China; 4 Department of Medicine and Health Sciences, Linköping University, 581 83. Linköping, Sweden; 5 Zhejiang First affiliated Hospital of Chinese Medical University, Hangzhou, 310006, People´s Republic of China; 6 Department of Cardiovascular Sciences, University of Leicester and NIHR Leicester Cardiovascular Biomedical Research Unit, Glenfield Hospital, Leicester, LE3 9QP, UK

## Abstract

Retinoblastoma is a highly invasive malignant tumor that often invades the brain and metastasizes to distal organs through the blood stream. Invasiveness and metastasis of retinoblastoma can occur at the early stage of tumor development. However, an optimal preclinical model to study retinoblastoma invasiveness and metastasis in relation to drug treatment has not been developed. Here, we developed an orthotopic zebrafish model in which retinoblastoma invasion and metastasis can be monitored at a single cell level. We took the advantages of immune privilege and transparent nature of developing zebrafish embryos. Intravitreal implantation of color-coded retinoblastoma cells allowed us to kinetically monitor tumor cell invasion and metastasis. Further, interactions between retinoblastoma cells and surrounding microvasculatures were studied using a transgenic zebrafish that exhibited green fluorescent signals in blood vessels. We discovered that tumor cells invaded neighboring tissues and blood stream when primary tumors were at the microscopic sizes. These findings demonstrate that retinoblastoma metastasis occurs at the early stage and antiangiogenic drugs such as *Vegf* morpholino and sunitinib could potentially interfere with tumor invasiveness and metastasis. Thus, this orthotopic retinoblastoma model offers a new and unique opportunity to study the early events of tumor invasion, metastasis and drug responses.

Retinoblastoma is a genetically related malignancy that occurs as the most common ocular tumor in a population of the early-age children^1^. Inactivation of the retinoblastoma gene (Rb) in both alleles is responsible for the etiology owing to dysfunction of the Rb tumor suppressor gene^2^,^3^. Due to the young age and modest symptoms, retinoblastoma is usually diagnosed at the late stage of tumor development. Surgical enucleation is a standard approach for unilateral retinoblastoma and preservation of the better eye is often recommended for treatment of bilateral cases. For more advanced diseases, radiation and chemotherapy are required in addition to enucleation. Untreated retinoblastoma usually leads to a fatal consequence. High dose chemotherapy together with stem cell rescue offers an alternative therapeutic option for treatment of advanced and metastatic retinoblastoma. High dose of chemotherapy often causes broad toxic effects. Therefore, early diagnosis and the control of tumor progression are the key determinants for better prognosis.

Retinoblastoma often exhibits an invasive and metastatic phenotype at the early stage of tumor development^1^. The most common route of invasive spread is along the optic nerve to the brain, where tumors can further metastasize to other organs^4^. Additionally, tumors can also invade adjacent tissues including bone, orbital tissue, and the nasopharyngeal region via the sinus. Invasion of the optic nerve and subsequent spreading to the circulating subarachnoid fluid that further carry tumor cells to the spinal cord is an alternative pathway of metastasis. Similar to other solid tumors, retinoblastoma often disseminates into the blood circulation and further metastasizes to remote tissues and organs. Despite lacking lymphatics in the eye and orbit, massive extraocular invasion can also result in cancer spread into the lymphatic system.

Preclinical retinoblastoma models are mainly developed in mice owing to the availability of genetic tools in this experimental species^2^,^4^. Consequently, several lines of transgenic mouse models are available in the scientific community. However, these genetically manipulated mouse retinoblastomas often carry overexpression of a particular oncogene such as SV40-T antigen or loss of a tumor suppressor gene such as p53^5^. These oncogene-driven models are far from clinical relevance as activation of oncogenes and inactivation of tumor suppressor genes may not exist in human retinoblastomas. For example, SV40-T antigen is not present in human retinoblastomas. Our present work reports an orthotopic model that allows visualization of retinoblastoma invasion and metastasis at the single cell level. Moreover, the retinoblastoma development occurs at the early age of zebrafish development and thus recapitulates the pediatric situation in human patients. Importantly, our zebrafish retinoblastoma model offers a unique opportunity to study the mechanisms underpinning metastasis and to assess therapeutic efficacies of drugs that block retinoblastoma invasion.

## Results

### An invasive model of retinoblastoma

To recapitulate the clinical situation of retinoblastoma development, we developed an embryonic zebrafish model that would fulfill the following criteria: 1) Developing zebrafish to resemble the pediatric situation in human patients; 2) Immune privilege to allow implantation of human and mouse retinoblastoma tumors; 3) Orthotopic implantation to recapitulate the clinical origin of retinoblastoma; 4) Transparent visualization of implanted primary and metastatic tumors at the single cell level; 5) Quantitatively monitoring and assessing tumor cell behavior in the living body of zebrafish; 6) Interaction between retinoblastoma cells and host structural and cellular components in a non-invasive manner; and 7) Assessment of therapeutic effects of drugs that interfere with tumor invasion and metastasis.

**Figure 1:**
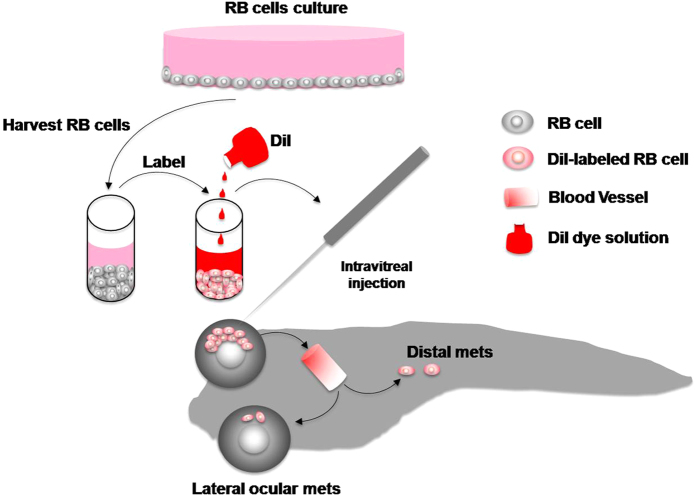
Schematic diagram of the orthotopic retinoblastoma model in zebrafish. Human and mouse retinoblastoma cells are cultured in RPMI-1640 and subconfluent tumor cells are harvested and labeled with DiI dye (red). Labeled tumor cells were intravitreally implanted in a single eye of each zebrafish. Tumor cell invasion, dissemination and distal metastasis can be visualized under fluorescent microscopy in the body of living zebrafish.

To achieve these aims, we labeled human or mouse retinoblastoma cells with the cell membrane dye DiI^6^,^7^ (Fig. 1). Approximately, 150 DiI-labeled retinoblastoma cells were intravitreally injected into the eye of each zebrafish. The formation of primary tumor and invasion as well as metastasis of injected tumor cells could be easily monitored by multi-channeled fluorescent microscopy (Fig. 1). In the living zebrafish body, retinoblastoma metastasis in the contralateral eye and in remote organs could be detected at the single cell level. The assay could be easily performed and the results were highly reproducible.

### Kinetic monitoring of primary retinoblastoma in zebrafish

**Figure 2:**
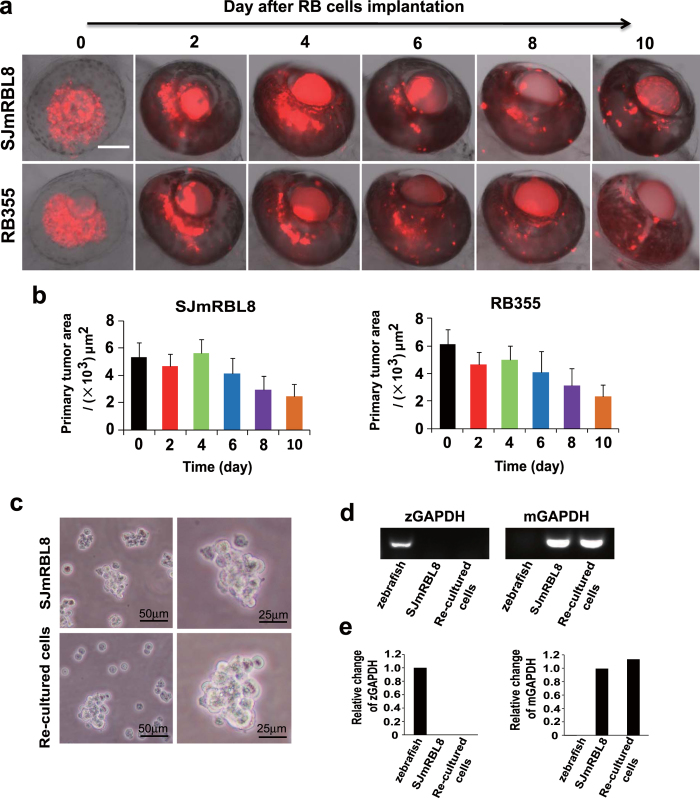
Formation of primary retinoblastoma in the zebrafish retina, and re-culture of implanted retinoblastoma cells from zebrafish *in vitro*. (**a**) Approximately, 150 DiI-labeled human (RB355) or mouse (SJmRBL-8) retinoblastoma cells (red color) were intravitreally implanted into each eye of zebrafish and the formation of primary tumors were kinetically monitored under fluorescent microscopy at different time points after tumor cell implantation. Bar = 100 μm. (**b**) Primary tumor areas were quantified using a digital Photoshop program. Approximately 30–40 tumor samples were used for quantification. (**c**) Transplanted SJmRBL-8 retinoblastoma cells are successfully isolated and cultured *in vitro*. The photographs at day 14 from inverted microscope showed the transplanted cells maintained their morphology and viability after injection. (**d**) Total RNA was extracted from zebrafish embryos, mouse SJmRBL-8 cells and the re-cultured cells, and RT-PCR was then used to analyze zebrafish and mouse GAPDH expression. (**e**) The expression of zebrafish and mouse GAPDH was determined by quantitative real-time PCR.

To develop an orthotopic retinoblastoma model, we chose human invasive RB355 retinoblastoma cell line and non-invasive WERI-Rb1 cell line, and mouse SJmRBL-8 invasive cell line for tumor implantation^4^,^8^,^9^,^10^. After implantation, tumor growth and invasion could be kinetically monitored at different time points. At day 0 of implantation of mouse SJmRBL-8 and human RB355 retinoblastoma cells, the injected tumor cells formed clusters that reassembled a primary tumor mass (Fig. 2a). At day 2 after implantation, these clusters of tumor cells became more packed tumor colonies and increased densities of tumor masses were observed at day 4 after tumor implantation (Fig. 2a). However, spreading of tumor cells were markedly increased along embryonic development after day 6 of tumor implantation. At day 10 after implantation, a spreading pattern of primary tumors was observed in both human and mouse retinoblastomas (Fig. 2a). Quantification analyses showed that the primary tumor masses of human and mouse retinoblastomas were progressively decreased along zebrafish development (Fig. 2b). These data indicate that primary human and mouse retinoblastomas lacked the ability of growth, but retained their invasive features.

### *In vitro* re-culturing of viable retinoblastoma cells from implanted zebrafish

To exclude the possibility the implanted retinoblastoma cells were dead, we isolated the implanted retinoblastoma cells from the tumor-implanted zebrafish embryos at day 4 after implantation. Noticeably, the implanted SJmRBL-8 retinoblastoma cells in zebrafish eyes were successfully re-cultured. Detectable tumor cell colonies were readily detected after day 5 incubation in cell culture and large tumor colonies were formed after 2-week incubation (Fig. 2c). The re-cultured tumor cells maintained their original morphologies, which was indistinguiable from the original SJmRBL-8 retinoblastoma cells (Fig. 2c). In order to further confirm the re-cultured cells as the implanted retinoblastoma cells rather than zebrafish original cells, we extracted the RNA from zebrafish embryos, mouse SJmRBL-8 cells and the re-cultured cells, respectively. The results from RT-PCR and quantitative real-time PCR both showed that zebrafish GAPDH was not expressed in mouse SJmRBL-8 cells and the re-cultured cells, while the re-cultured cells expressed mouse GAPDH, exactly as mouse SJmRBL-8 cells (Fig. 2d,e). These results indicate that the implanted retinoblastoma cells were still alive and maintained their morphology and probably invasive features as the original retinoblastoma cells in our zebrafish model.

### Invasiveness and metastasis at the early stage of tumor development

**Figure 3:**
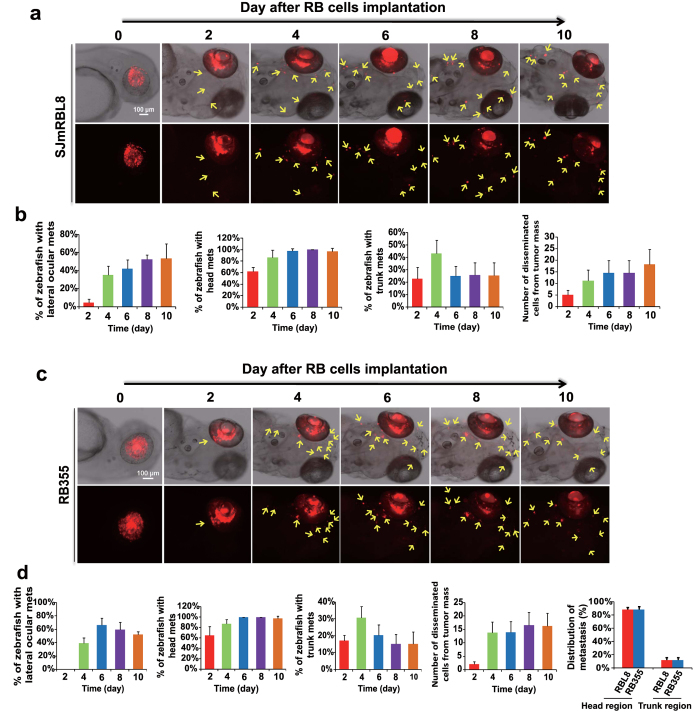
Retinoblastoma invasion and metastasis in the zebrafish (**a**) Time-course monitoring invasion and metastasis of DiI-labeled mouse SJmRBL-8 retinoblastoma cells (red color) in the head region of zebrafish embryos. Arrows indicate metastatic tumor cells in single cell or a cluster cells. Bar = 100 μm. (**b**) Quantification of percentage of zebrafish embryos with contralateral ocular SJmRBL-8 metastasis, percentage of zebrafish embryos with head metastasis, percentage of zebrafish embryos with trunk metastasis, and the average total number of metastatic tumor cells per zebrafish (n = 40 embryos/group). (c) Time-course monitoring invasion and metastasis of DiI-labeled human RB355 retinoblastoma cells (red color) in the head region of zebrafish embryos. Arrows indicate metastatic tumor cells in single cell or a cluster cells. Bar = 100 μm. (**d**) Quantification of percentage of zebrafish embryos with contralateral ocular RB355 metastasis, percentage of zebrafish embryos with head metastasis, percentage of zebrafish embryos with trunk metastasis, the average total number of metastatic tumor cells per zebrafish, and the distribution of metastatic SJmRBL-8 and RB355 tumor cells in head and trunk regions at day 4 after primary tumor implantation (n = 40 embryos/group).

**Figure 4:**
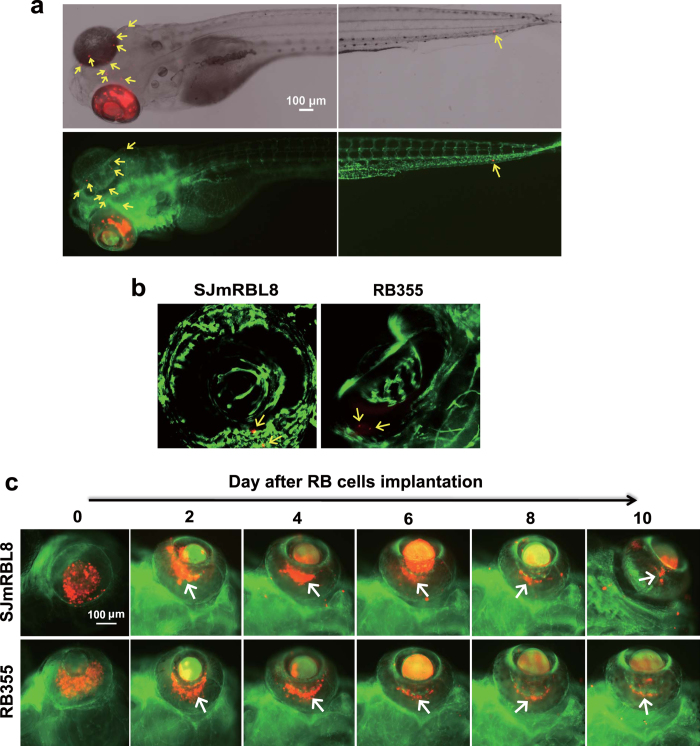
Interaction between retinoblastoma cells and vasculatures (**a**) A representative image of the entire fli1:EGFP zebrafish embryo that received SJmRBL-8 retinoblastoma cell implantation at day 4. The left eye was implanted with DiI-labeled tumor cells (red) and zebrafish vasculatures were visualized by expression of EGFP. Arrows point to the metastatic tumor cells in the head and trunk regions. Bar = 100 μm. (**b**) Detection of DiI-labeled metastatic retinoblastoma cells in the contralateral eye by confocal imaging analysis. (**c**) Visualization of implanted retinoblastoma cells in relation to hyaloid vasculatures at different points after tumor cell implantation. Arrows indicate the position of primary tumors growing in tight association with hyaloid vessels. Bar = 100 μm.

Knowing invasiveness and metastasis of retinoblastoma occur at the early stage of tumor development, we investigated the metastatic process in this model in great details. Kinetic studies showed that dissemination of tumor cells from the primary sites occurred at day 2 after human RB355 and mouse SJmRBL-8 tumor cells implantation (Fig. 3). Total numbers of metastatic foci was progressively increased and reached the maximal level at day 6 after tumor implantation. Interestingly, approximately 90% of metastatic tumor cells were located in the head region of the zebrafish body, suggesting that cancer metastasis along the optic nerve and local invasion might be dominant metastatic pathways of retinoblastoma spreading. Given the fact that retinoblastoma in human patients commonly metastasize along optic nerve and through local invasion in the head region, our zebrafish model is highly relevant to the clinical situation. The existence of a similar metastatic pattern of SJmRBL-8 and RB355 retinoblastomas further supported that the clinical relevance of this preclinical model. In the head region, we also found that a substantial number of tumor cells metastasized to the contralateral healthy eye (Figs 3,4a). The existence of contralateral metastatic retinoblastoma cells was further validated by confocal microscopy analysis (Fig. 4b). Additionally, retinoblastoma cells were occasionally detected in remote parts of the body such as the truck region of the zebrafish body, indicating the existence of a hematogenous mechanism of cancer metastasis in zebrafish bodies (Fig. 4a). However, implantation of human non-invasive WERI-Rb1 tumor cells did not result in dissemination in our zebrafish model (Figure S1). This is consistent with previous study^4^. Taken together, our zebrafish retinoblastoma model demonstrates that metastasis occurs at the early stage of tumor development and metastatic modules are mainly distributed in the head region of the zebrafish body, thus recapitulating the clinical situation of retinoblastoma invasiveness.

### Vascular roles in primary retinoblastoma and metastasis

Blood vessels are essential for primary tumor survival, growth and invasiveness^11^. We next studied the interaction between retinoblastoma cells and the retinal vasculature. For this reason, we used the *fli1:EGFP* zebrafish strain that expresses enhanced green fluorescent protein in all vascular endothelial cells^6^,^12^,^13^,^14^,^15^. At the 48 hours post fertilization (hpf), zebrafish embryos develop the hyaloid vasculature that extends from the retina to the lens. At day 2 after tumor cell implantation, we have found that most tumor cells formed clusters around the hyaloid vessels attached to lens (Fig. 4c). Association of retinoblastoma cells with hyaloid vessels was enhanced from day 2 to day 4 post-tumor implantation and tumor masses became decreased thereafter (Fig. 4c). These findings suggest that the retinal vasculature provides a structure basis of tumor formation and probably tumor invasion.

### *Vegf-aa* morpholino and Sunitinib treatment attenuates retinoblastoma metastasis

**Figure 5:**
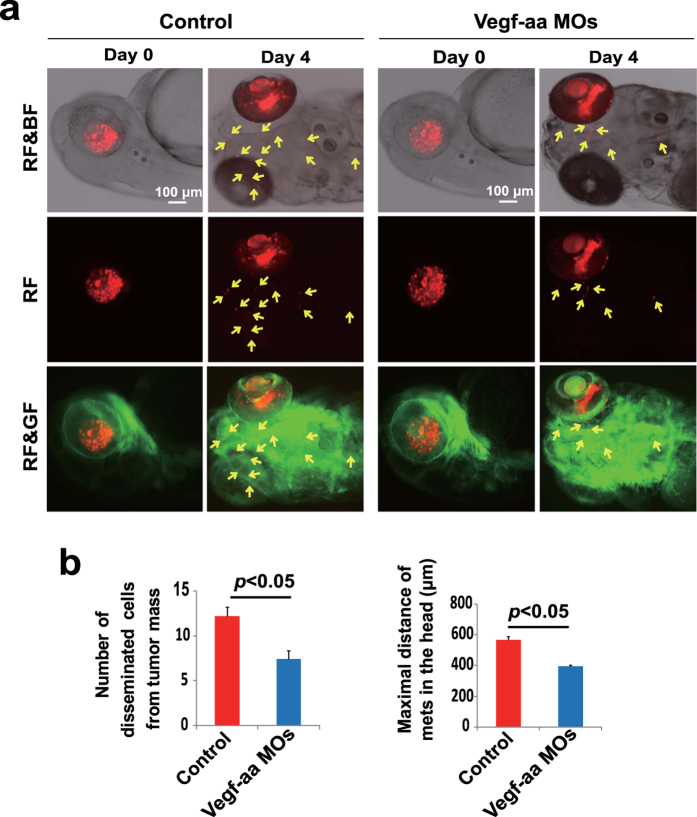
*Vegf-aa*morpholino treatment attenuates retinoblastoma metastasis. (**a**) *Vegf-aa* specific morpholinos and control morpholinos were injected into the yolk of each embryo at the 1-2 cell stage. DiI-labeled SJmRBL-8 retinoblastoma cells were intravitreally implanted in zebrafish at 48 hpf. Retinoblastoma invasion and metastasis were monitored at day 0 and day 4 post-implantation. Arrows point to metastatic tumor cells. Bar = 100 μm. (**b**) Quantification of metastatic tumor cells and the averages of maximal distance of metastatic foci in *Vegf-aa* and control morpholinos-treated zebrafish embryos (n = 20 embryos/group).

As vasculatures plays a crucial role in tumor invasion and metastasis and VEGF is an essential mediator for tumor angiogenesis and vascular remodeling, we studied the function of VEGF using a specific morpholino targeting zebrafish *Vegf-aa* mRNA as previously described^14^. Expectedly, delivery of *Vegf-aa* morpholino to tumor-bearing zebrafish embryos effectively inhibited tumor cell dissemination to distal regions (Fig. 5a,b). These findings show that VEGF-modulated vessel growth and structural changes are crucial for retinoblastoma metastasis.

**Figure 6:**
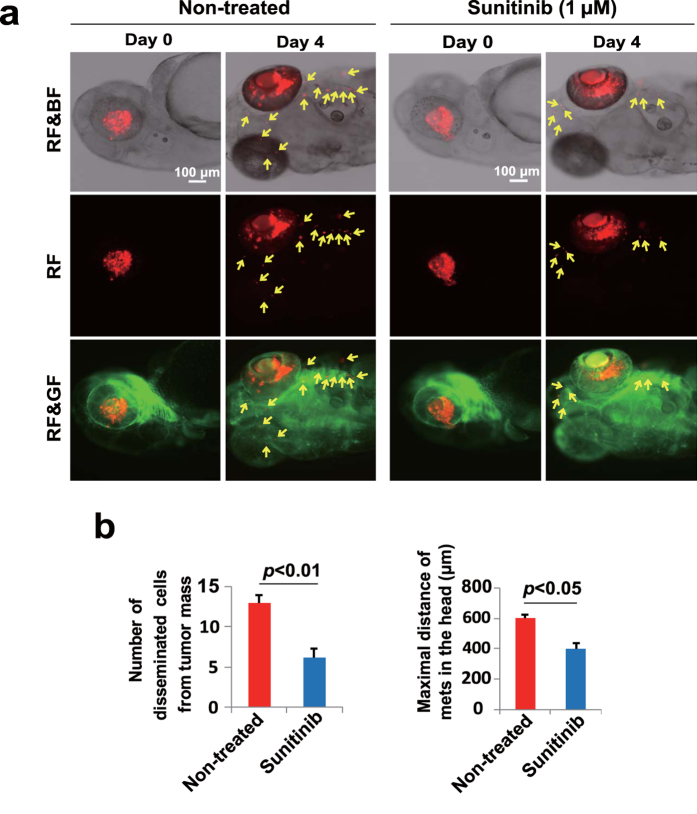
Sunitinib inhibits mouse retinoblastoma invasion and metastasis. (**a**) SJmRBL-8 retinoblastoma cells were intravitreally implanted in zebrafish and sunitinib was added to the aquarium water to constitute a final concentration of 1 μM. Retinoblastoma invasion and metastasis were monitored at different time points. Arrows point to metastatic tumor cells. Bar = 100 μm. (**b**) Quantification of metastatic tumor cells and the averages of maximal distance of metastatic foci in vehicle- and sunitinib-treated zebrafish embryos (n = 60 embryos/group).

The current retinoblastoma model in zebrafish might provide an excellent opportunity for therapeutic interference with drugs. For this reason, we chose sunitinib that blocks signaling pathways of VEGFRs and other receptor kinases^16^,^17^. The advantage of choosing sunitinib is that this antiangiogenic drug is orally active and could be administrated in the aquarium water^15^. Treatment with retinoblastoma-bearing zebrafish embryos with 1 μM of sunitinib significantly attenuated mouse and human retinoblastoma cells invasion and metastasis (Fig. 6 and Figure S2). It should be emphasized that sunitinib is a broad tyrosine kinase inhibitor that blocks signal transduction of other kinase activity, which may not be related to angiogenesis. Given the fact that retinoblastoma often invades and metastasizes along optic nerve, it is likely sunitinib could also affect this pathway of metastasis. Nevertheless, anti-metastatic effect of retinoblastoma by sunitinib provides a conceptual principle for potential treatment of retinoblastoma invasion and metastasis by targeted therapeutic that block tumor angiogenesis and other signaling pathways.

## Discussion

Optimal animal models that recapitulate the onset, progression and metastasis of retinoblastoma in human patients are crucial for understanding the mechanisms underlying this malignant disease and for development of effective therapies. Although several mouse retinoblastoma models have been developed^4^,^5^, these mouse-based cancer models are often driven by activation of a particular oncogene or inactivation of a tumor suppressor gene, which may not reflect the intrinsic features of retinoblastoma in human patients. For example, the SV40-T antigen-driven retinoblastoma model^19^ would artificially activate particular oncogenic pathways that do not exist in human patients. Consequently, effective new drugs evaluated based on this preclinical model may be ineffective in human patients.

Similar to most other solid malignant tumors, retinoblastoma invasion and metastasis may occur at the early stage of cancer development. Intimate interactions between tumor cells and the cellular components in the tumor microenvironment exist at the early time point when tumor cells are formed as microscopic lesions. Microscopic tumors often form manacles around microvessels from which nutrients and oxygen can be nourished by the mechanism of free-diffusion^20^,^21^. However, intimate interactions between tumor cells and microvessels are likely to facilitate dissemination of malignant cells from the primary sites. This is particularly true for those cancer patients who are initially diagnosed for cancer disease based on metastatic lesions whereas their primary tumors are not detectable. Perhaps tumors at the primary site lack the capacity of switching to an angiogenic phenotype^22^ and thus are unable to grow into a detectable size. In the case of retinoblastoma, this tumor type is originated from the retina that contains an exceptionally high density of microvessels^23^. Thus tumor development occurs concomitantly with vascular development in the retina. In young children, retinal vasculatures might exhibit specific features such as remodeling and maturation, which would be potentially susceptible for tumor cell invasion. This possibility has not been investigated experimentally.

An orthotopic retinoblastoma zebrafish model has been developed in the previous study^24^. It has been reported that transplanted retinoblastoma cells stably maintain in the vitreous cavity of zebrafish after the injection and systemic application of chemotherapies can reduce tumor population^24^. However, tumor metastasis has not been reported in that study. In our orthotopic retinoblastoma zebrafish model, we demonstrated retinoblastoma tumor cells begin to invade and disseminate at the early stage of tumor implantation. Zebrafish embryos lack pigmentation, which allows visualization of tumor cell migration and invasion. Detection of tumor cells can be achieved at the single cell level^6^. Therefore, our model provides a unique opportunity to investigate the early events of cancer metastasis. Intravasation into the circulation is one of the initial processes of dissemination of malignant cells to distal tissues and organs. Using the *fli1:EGFP* transgenic zebrafish, the detailed process of intravasation of retinoblastoma cells could be studied. Additionally, the availability of other genetically manipulated zebrafish strains may offer many opportunities to study other cellular and molecular components in cancer metastasis in the tumor microenvironment. These other opportunities warrant further investigation. In addition to zebrafish models, a recent study shows that human retinoblastoma cells implanted into the vitreous of newborn rat eyes offer an attractive mammalian orthotopic model to study the spatiotemporal development of retinoblastoma^25^. With bioluminescence imaging and optical coherence tomography analyses, tumor growth in relation to vascularization can be studied in details. This rat model in combination with our current zebrafish model would be complementary and reveal some important information of retinoblastoma growth, invasion and metastasis.

Treatment and prevention of invasion and metastasis are the key determinants for survival improvement in retinoblastoma patients. In the present study, we show that sunitinib treatment significantly inhibited the invasive and metastatic phenotypes of retinoblastoma in zebrafish. The mechanisms that underlie the anti-metastatic effects of sunitinib may include: 1) inhibition of VEGF-induced angiogenesis at the primary tumor site; 2) inhibition of VEGF-induced vascular leakage, which would otherwise increase susceptibility of tumor cell invasion; and 3) inhibition of non-VEGF-related invasive pathways both in tumor cells and in host cells as this drug targets multiple signaling pathways. Despite involvement of multiple mechanisms, sunitinib treatment effectively block retinoblastoma invasion in our model system. If these findings can be successfully translated into clinical practice, treatment of retinoblastoma with sunitinib and perhaps other antiangiogenic targeted therapeutics would offer a new therapeutic option for treatment of retinoblastoma in human patients. Taken together, our present work has provided new mechanistic insights of retinoblastoma invasion and metastasis and has paved a new avenue for therapeutic intervention of this invasive disease by targeting angiogenesis.

## Methods

### Cell culture and preparation for implantation

Mouse SJmRBL-8 and human RB355 retinoblastoma cell lines were kindly provided by Dr. Michael A. Dyer at St. Jude children’s research hospital, Memphis, Tennessee, USA. Human WERI-Rb1 cell line was obtained from the American Type Culture Collection (Manassas, VA, USA). The original RB355 human retinoblastoma cell line was generated from a non-familial and unilaterally affected 11-month-old child. This cell line was originally established by Dr. Brenda Gallie (University of Toronto)^9^ and contains genetic mutations of the Rb gene^26^. Pathological findings showed that retinoblastoma cells invaded the choroid and optic nerve when the patient had enucleation surgery^9^. SJmRBL-8 was originally established from the Rb^Lox/+^ transgenic retinoblastoma mouse strain by Dr. Michael A. Dyer at St. Jude Children’s Research Hospital, Memphis, Tennessee. It has been described as highly invasive tumor type in mouse tumor models^10^. Human WERI-Rb1 retinoblastoma cell line was originally established by Dr. McFall (Wills Eye Research Institute)^27^, and it has been reported as a non-invasive and metastatic tumor^4^. These retinoblastoma cells were grown and maintained in RPMI-1640 medium supplemented with 10% fetal bovine serum (FBS, cat. no.SV30160.03; Hyclone), 1% L-glutamine, and 1% penicillin-streptomycin. Tumor cells were labeled with 1,1′-dioctadecyl-3,3,3′,3′-tetramethylindocarbocyanine perchlorate (DiI, cat. no. D3899; Invitrogen) according to our previously described method^6^. Briefly, tumor cells grown at the log phase were washed twice with PBS, followed by labeling with a final concentration of 2 μg/ml of DiI for 30 min at 37 °C. After rigorous washing with PBS, tumor cells were further cultured overnight.

### Intravitreal implantation of retinoblastoma cells in zebrafish embryos

All experimental procedures of our zebrafish study conformed to the Association for Research in Vision and Ophthalmology Statement for the Use of Animals in Ophthalmic and Vision Research. Our study was approved by the Northern Stockholm Experimental Animal Ethical Committee. The *fli1:EGFP* transgenic zebrafish were used for some experiments^12^. Fertilized zebrafish eggs were collected and incubated at 28.5 °C in the Danieau’s solution. At 24 hours post fertilization (hpf), zebrafish embryos were transferred into water containing 0.2 mM 1-phenyl-2-thio-urea (PTU, cat. no. P7629; Sigma-Aldrich) to inhibit pigmentation. At 48 hpf, zebrafish embryos were dechorionated with a forceps and anesthetized with 0.04 mg/ml tricaine (MS-222, Sigma-Aldrich) before receiving tumor cell injection. Zebrafish embryos were subsequently transferred onto a 2% agarose gel. DiI-labeled tumor cells were counted under a phase contrast microscope, centrifuged at 1000 rpm for 5 min, and resuspended at a final concentration of 30–40 cells per nl in 0.1% FBS-RPMI-1640 medium. Approximately, 100–200 retinoblastoma cells were injected into the vitreous cavity of zebrafish embryo with non-filamentous borosilicate glass capillary needles attached to the microinjector under the stereomicroscope (Leica Microsystems). A single eye was injected with tumor cells in each zebrafish embryo. After tumor cell injection, zebrafish embryos were further selected under fluorescent microscopy to ensure that tumor cells were located only within the eye balls, and then incubated in aquarium water for consecutive ten days at 28.5 °C.

### Isolation and culturing the implanted retinoblastoma cells from zebrafish embryos

At day 4 after retinoblastoma cells implantation, 50 implanted zebrafish embryos were collected and euthanized with a lethal dose of tricaine. Zebrafish embryos were transferred to a culture plate filled with RPMI-1640 medium supplemented with 10% FBS and 1% penicillin-streptomycin. The tumor-implanted zebrafish eyes were enucleated under a dissecting microscope. The cell suspension containing the fragments of eyes was filtered with a 100 μm filter. The isolated cells were collected and suspended in a complete RPMI-1640 medium with 10% FBS, and seeded in a 96-well plate. Tumor cell colonies were detected at day 5 after culturing and photographed at day 14.

### RT-PCR and quantitative real-time PCR to verify the re-cultured cells as original implanted cells

Total RNA isolated from zebrafish embryos, mouse SJmRBL-8 cells and the re-cultured cells was used for RT-PCR and quantitative real-time PCR analysis. Briefly, 100 ng total RNA from each sample was reverse-transcribed using a Revert Aid H minus First Strand cDNA Synthesis Kit (Fermentas). Reverse transcription was performed at 42 °C for 60 min, followed by 70 °C for 5 min to inactivate the enzyme activity. RT-PCR was performed in a 20 μl reaction mixture containing Dream Taq Green PCR Master Mix (Thermo Fisher Scientific), 500 nM forward and reverse primers, and 1 μl cDNA. The RT-PCR protocol was executed for 28 cycles, and each cycle consisted of denaturation at 95 °C for 30 s, annealing at 60 °C for 30 s, and extension at 72 °C for 1 min. Quantitative real-time PCR was performed in a duplicate and each 20 μl reaction contained SYBR Green (Applied Biosystems), 150 nM forward and reverse primers, and 1 μl of cDNA using an ABI Prism 7500 System (Applied Biosystems). Quantitative real-time PCR protocol was executed for 40 cycles and each cycle consisted of denaturation at 95 °C for 15 s, annealing at 60 °C for 1 min, and extension at 72 °C for 1 min. The primer pairs specific for various genes used in our experiments include: zebrafish GAPDH forward, 5'-TAACGGATTCGGTCGCATTG-3'; zebrafish GAPDH reverse, 5'-GGCTGGGTCCCTCTCGCTA-3'; mouse GAPDH forward, 5'-CCAGCAAGGACACTGAGCAA-3'; mouse GAPDHreverse, 5'-GGGATGGAAATTGTGAGGGA-3'.

### Fluorescent imaging analysis

Primary tumor growth, invasion and metastasis in the zebrafish body were monitored every other day with a fluorescent microscope (Nikon Eclipse C1). Each zebrafish embryo was picked up with a Pasteur pipette and was carefully placed onto the agarose cushion embedded on a glass slide. Excessive water was removed and a small drop of 0.04% tricaine was added onto the zebrafish embryo-containing water. The entire zebrafish body was monitored to detect tumor cell distribution. The interaction between tumor cells and surrounding microvessel vasculature was detected using a double-color system. Typically, two different sets of images from the head region and the trunk region were collected separately from each zebrafish embryo. Disseminated tumor cells in each eye of zebrafish embryos were counted and the primary tumor areas were measured using Adobe Photoshop CS software. At least 20–30 embryos were included in each experimental group.

### *Vegf-aa* morpholino treatment

Morpholino oligonucleotide (MOs) was obtained from Gene tools (Philomath, OR, USA). *V*egf*-*aa-MO (5′-GTATCAAATAAACAACCAAGTTCAT-3′) and standard scrambled control-MO (5′-CCTCTTACCTCAGTTACAATTTATA-3′) have been used in our previous publication^14^. They were prepared at 3 mM stocks and diluted in nuclease-free water. Approximately 1 nl of 0.06 pM was injected into the yolk of each embryo at the 1-2 cell stage. The injected embryos were then placed in fresh Danieu's medium or E3 medium, followed by injecting retinoblastoma cells at 48 hpf indicated above.

### Sunitinib treatment

Before treatment, sunitinib powder was dissolved in the distilled water to achieve the concentration of 1 mM stock solution. After tumor cells implantation, the stock solution was added directly to the aquaria water to attain a final concentration of 1 μM, and zebrafish embryos were incubated for 4 days. At day 4 after treatment, the dissemination and invasion of the tumor cells were examined with a fluorescent microscopy. In addition, to quantitative analyze the distance of metastasis, choose the cell that lies furthest away from the primary tumor mass and measure the distance between that cell and the primary tumor using Adobe Photoshop CS software. At least 13–20 embryos in each group were used to obtain statistically significant values.

### Confocal microscopy imaging

Zebrafish embryos were euthanized with a lethal dose of tricaine. The heads of zebrafish were separated and mounted in vectashield mounting medium (cat. no. H-1000; Vector Laboratories) onto the glass slides for confocal analysis. The eyes of zebrafish were visualized with a confocal microscope (Nikon EZ-C1) using three-dimensional (3-D) scanning at high magnification (×40 objectives). To obtain high-quality 3-D images, each eye was scanned in 8–10 layers, which were assembled to constitute one dataset using a confocal microscope software (EZ-C1).

### Statistical Analysis

Experimental data were presented as mean determinants ± SEM and analyzed using a two-tailed Student *t*-test. Statistical *P* values were presented as follows: *P* < 0.05 was significant; and *P* < 0.01 was highly significant.

## Additional Information

**How to cite this article**: Chen, X. *et al.* Invasiveness and metastasis of retinoblastoma in an orthotopic zebrafish tumor model. *Sci. Rep.* **5**, 10351; doi: 10.1038/srep10351 (2015).

## Supplementary Material

Supplementary Information
