# Supplementary material for: Invasiveness and metastasis of retinoblastoma in an orthotopic zebrafish tumor model
Source: Sci Rep. 2015 Jul 14;5:10351. doi: 10.1038/srep10351 (PMC4501005; doi:10.1038/srep10351)
Supplement: Supplementary Information [file srep10351-s1.doc]

**Supplementary information**

**Invasiveness and metastasis of retinoblastoma in an orthotopic zebrafish tumor model**

Xiaoyun Chen^1,2♯^, Jian Wang^2,3♯^, Ziquan Cao^2,4^, Kayoko Hosaka^2^, Lasse Jensen^2,4^, Huasheng Yang^1^, Yuping Sun^3^, Rujie Zhuang^5^, Yizhi Liu^1*^ and Yihai Cao^2,4,6*^

^1^The State Key Laboratory of Ophthalmology, Zhongshan Ophthalmic Center, Sun Yat-Sen University, Guanzhou, 510060, People´s Republic of China

^2^Department of Microbiology, Tumor and Cell Biology, Karolinska Institute, 171 77 Stockholm, Sweden

^3^Department of Oncology, Jinan Central Hospital, Shandong University, NO.105, Jiefang Road, Jinan, Shandong 250013, People's Republic of China.

^4^Department of Medicine and Health Sciences, Linköping University, 581 83. Linköping, Sweden

^5^Zhejiang First affiliated Hospital of Chinese Medical University, Hangzhou, 310006, People´s Republic of China

^6^Department of Cardiovascular Sciences, University of Leicester and NIHR Leicester Cardiovascular Biomedical Research Unit, Glenfield Hospital, Leicester, LE3 9QP, UK.

*Correspondence: Yihai Cao, M.D., Ph.D., Department of Microbiology, Tumor and Cell Biology, Karolinska Institutet, 171 77 Stockholm, Sweden. Tel: (+46)-8-5248 7596, Fax: (+46)-8-33 13 99, E-mail: [yihai.cao@ki.se](mailto:yihai.cao@ki.se); Or Yizhi Liu, M.D., Ph.D., The State Key Laboratory of Ophthalmology, Zhongshan Ophthalmic Center, Sun Yat-Sen University, 510060 Guanzhou, People´s Republic of China. Tel: (+86)-020-87330293, Fax: +86-020-87333271, Email: [yizhi_liu@aliyun.com](mailto:yizhi_liu@aliyun.com).

^♯^These authors contributed equally to this work.

**Figure S1**

**Figure S2**

**Figure legends**

**Figure S1 | Formation of primary retinoblastoma, invasion and metastasis of non-invasive retinoblastoma cells in the zebrafish.** Approximately 150 DiI-labeled human non-invasive WERI-Rb1 retinoblastoma cells (red color) were intravitreally implanted into the eye of zebrafish embryo. The formation of primary tumors, and invasion and metastasis were kinetically monitored under fluorescent microscopy at different time points after tumor cell implantation. Approximately 30-40 zebrafish were used for this experiment.

**Figure S2 | Sunitinib inhibits human retinoblastoma invasion and metastasis.** (a) RB355 retinoblastoma cells were intravitreally implanted in zebrafish and sunitinib was added to the aquarium water to constitute a final concentration of 1 μM. Retinoblastoma invasion and metastasis were monitored at different time points. Arrows point to metastatic tumor cells. Bar = 100 μm (b) Quantification of metastatic tumor cells and the averages of maximal distance of metastatic foci in vehicle- and sunitinib-treated zebrafish embryos (n = 60 embryos/group).
